# Developmentally dynamic genome: Evidence of genetic influences on increases and decreases in conduct problems from early childhood to adolescence

**DOI:** 10.1038/srep10053

**Published:** 2015-05-06

**Authors:** Jean-Baptiste Pingault, Frühling Rijsdijk, Yao Zheng, Robert Plomin, Essi Viding

**Affiliations:** 1 King’s College London, MRC Social, Genetic and Developmental Psychiatry Centre, Institute of Psychiatry, London, United Kingdom; 2 Division of Psychology and Language Sciences, University College London, London, United Kingdom; 3 Department of Psychology, Simon Fraser University, Canada

## Abstract

The development of conduct problems in childhood and adolescence is associated with adverse long-term outcomes, including psychiatric morbidity. Although genes constitute a proven factor of stability in conduct problems, less is known regarding their role in conduct problems’ developmental course (i.e. systematic age changes, for instance linear increases or decreases).Mothers rated conduct problems from age 4 to 16 years in 10,038 twin pairs from the Twins Early Development Study. Individual differences in the baseline level (.78; 95% CI: .68-.88) and the developmental course of conduct problems (.73; 95% CI: .60-.86) were under high and largely independent additive genetic influences. Shared environment made a small contribution to the baseline level but not to the developmental course of conduct problems. These results show that genetic influences not only contribute to behavioural stability but also explain systematic change in conduct problems. Different sets of genes may be associated with the developmental course versus the baseline level of conduct problems. The structure of genetic and environmental influences on the development of conduct problems suggests that repeated preventive interventions at different developmental stages might be necessary to achieve a long-term impact.

The development of conduct problems in childhood and adolescence is associated with adverse long-term outcomes, such as increased mortality rate, psychiatric morbidity, and criminality^1^,^2^,^3^. Prevention of these long-term consequences by influencing the developmental course of conduct problems in high-risk children has proven challenging^2^,^4^. As such, a more in depth understanding of the source of individual differences in the developmental course of conduct problems from early childhood to adolescence is needed. In this longitudinal twin study, we aimed to examine environmental and genetic influences on the developmental course versus the baseline level of conduct problems from early childhood to adolescence.

Large meta-analyses of cross-sectional studies, pooling a range of measures of antisocial behaviour, have reported an overall moderate heritability of around 50%, with variations according to the type of measures of antisocial behaviour^5^,^6^,^7^. Studies focusing on conduct problems have reported similar or higher heritability estimates^8^,^9^. Longitudinal studies are necessary to examine whether these genetic factors underlying conduct problems are the same at different ages^10^,^11^. The extant genetically informative longitudinal studies of a range of measures of antisocial behaviour have found evidence for: 1) genetic continuity, i.e. the continuity in antisocial behaviour during childhood and adolescence is largely explained by genetic factors (with a small contribution of the shared environment); and 2) genetic innovation, i.e., despite genetic continuity, new genetic factors also emerge from childhood to adolescence^10^,^12^,^13^,^14^. The concept “genetic innovation” may seem confusing as the genetic material remains the same throughout life; however, genetic influences can manifest at the phenotypic level later in life, even for monogenic disorders such as the Huntington disease^15^. Similarly, genetic factors that were not expressed at an early age can start influencing conduct problems at a later age, pointing towards the importance of genetic influences in age-to-age change.

Although existing longitudinal studies show genetic influences on age-to-age change, they do not directly address the role of genes in the developmental course of conduct problems, i.e. systematic changes occurring with age, such as linear increases or decreases. Yet, this question is particularly relevant in the case of conduct problems. For instance, physically aggressive behaviour, a subset of conduct problems, emerges as early as the first year of life, is normative in early childhood, but decreases later on for most but not all children^16^,^17^,^18^. This suggests that physical aggression needs not to be learned whereas children may need to learn how not to use aggression^19^. Genetic factors may thus be largely responsible for individual differences in the baseline level of physical aggression and its continuity over time, whereas environmentally driven socialization processes might explain individual differences in the developmental course. However, genetically dependent neurodevelopmental processes may also influence the ability of children to progressively control their aggressive behaviour^5^,^20^, raising the possibility of genetic influences on its developmental course (i.e. genes not only explain continuity but also systematic change).

The respective role of genes and the environment in explaining the developmental course of conduct problems can be addressed by: 1) using a latent growth curve model to explicitly examine the baseline level (i.e. intercept) and the developmental course (e.g. systematic constant change such as a linear slope) of conduct problems; and 2) incorporating genetic and environmental influences on the latent structure, i.e. the intercept and the slope^21^. This model provides a direct estimate of the genetic and environmental contributions to the developmental course of conduct problems; it also enables the distinction between contributions specific to the developmental course and those shared with the baseline level. Using this model in a study of 667 twin pairs from age 20 to age 50 months, Lacourse *et al*.^22^ recently reported that the developmental course of physical aggression in early childhood was strongly influenced by genetic factors with no significant environmental influences. Interestingly, the genetic factors influencing the developmental course of physical aggression were totally different from those influencing the baseline level. This differential large genetic influence was labelled “genetic maturation” by the authors. Because physical aggression is only a subset of conduct problems and because new types of environmental influences (such as peer groups) may emerge in adolescence, it is important to evaluate this “genetic maturation” hypothesis beyond early childhood and into the key developmental period of adolescence. In addition, a much larger sample in the present study enables a finer examination of genetic and environmental influences.

This study using a large representative twin sample prospectively followed from early childhood to adolescence aimed to: 1) examine the genetic and environmental influences underlying the developmental course of conduct problems 2) verify whether these developmental influences were independent from or shared with those influencing the baseline level of conduct problems.

## Results

### Preliminary analyses

A total of 10,038 twin pairs from the Twins Early Development Study (TEDS) had one or more complete (i.e. both twins) assessment(s) of conduct problems between the ages of 4 and 16 years and were included in the study (see Methods and Supplementary Table 1). Table 1 shows the number of complete MZ and DZ twin pairs and twin correlations at each age (see Supplementary Table 2 for complete descriptive statistics). Consistent with meta-analyses of a range of measures of antisocial behaviour^5^,^7^, we detected no evidence for different genetic and environmental aetiology in males and females for the observed variables, so that sex differences were not considered any further in multivariate analyses. We fitted a standard Cholesky decomposition, as is commonly used in longitudinal studies^15^. Table 2 shows the total genetic and environmental components at each age. All genetic pathways were significant, showing genetic continuity at all ages. For instance, the genetic factors at age 4 years still explained 14% of the variance of conduct problems at 16 years (factor A1 to CP 16y in Table 2). The results also show substantial genetic innovation. For example, more than half of the genetic influence at age 16 years is independent of genetic influence at previous ages (Factor A4 to CP 16y in Table 2). Shared environmental influences were small and there was no evidence for innovation as shared environment influences at 7 and 12 years were mainly explained by the shared environment factor at 4 years. Non-shared environmental estimates (including measurement error) were mainly specific at each age, with no evidence for transmission from one age to another.

### Latent growth curve model (LGC)

**Figure 1:**
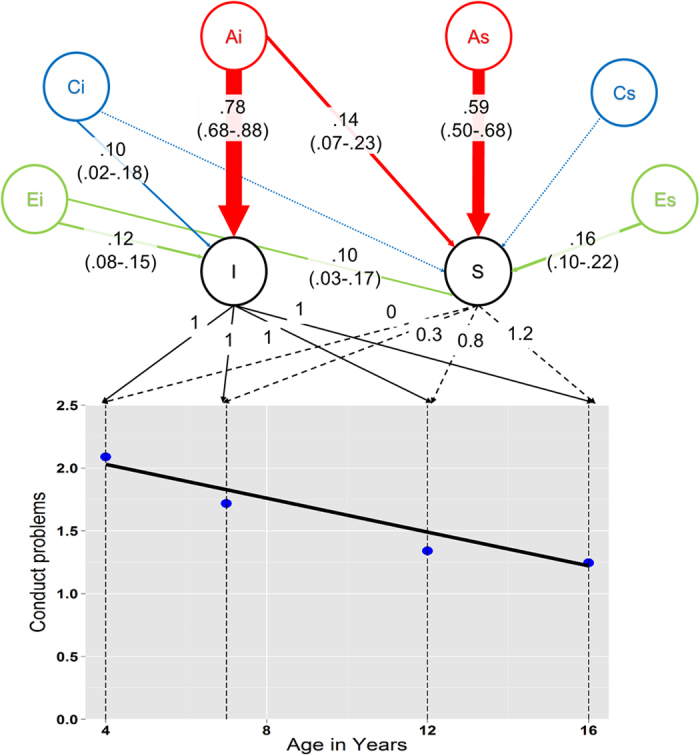
Genetic and environmental influences on the intercept and slope of conduct problems from age 4 years to age 16. Observed mean values of conduct problems (blue dots) and model fitted linear decrease (black line) are represented. The intercept (I) and the Slope (S) and their loadings are indicated (slope loadings equal distance in years from first measure, divided by 10 to facilitate computations). A (heritability), C (shared environment), E (non-shared environment) standardized components of variance and 95% bootstrapped confidence estimates are provided for I and S (except for the non-significant dotted lines). The width of the arrows is proportional to the effect. Dotted arrows represent non-significant effects.

A phenotypic LGC (i.e. without genetic decomposition) was fitted to determine the baseline level (i.e. intercept) and to test whether a linear slope was sufficient to account for the observed systematic change in conduct problem or if additional growth parameters (e.g. quadratic) were necessary. The initial linear phenotypic model fitted the data adequately as shown by approximate fit indexes in line with recommended cut-off values (see Supplementary Table 3). Both the intercept (2.04, *SE* = 0.01, p < .001) and the slope (-0.68, *SE* = 0.02, *p* < .001) were significant, meaning that conduct problems linearly decreased from an initial score of 2.04 at 4 years to 1.23 at 16 years (Fig. 1). There was a significant negative correlation between the intercept and the linear slope (standardized *r = *-0.49, *SE* = 0.02, *p* < .001). Both the variances of the intercept (1.42, *SE* = 0.03, *p* < .001) and the slope (0.71, *SE* = 0.04, *p* < .001) were significant, pointing towards important individual differences in the baseline level and the slope of conduct problems.

To explain these individual differences, the ACE components were then added to the linear model. ACE standardized variance components (i.e. percentages of the overall variance due to each component) for the baseline level and the slope are reported in Fig. 1. The baseline level of conduct problems was under high genetic influence (.78; 95% CI: .68-.88), meaning that 78% of the variance was explained by additive genetic influences. Small but significant shared and non-shared environment components were observed. The slope was also under high genetic influence (.73; 95% CI: .60-.86), with a significant non shared-environment component (.25; 95% CI: .15-.36) but no significant shared environment influence (.02; 95% CI: .00-.11). Fig. 1 shows that this total heritability (.73) of the slope is largely due to genetic factors specific to the slope (.59) rather than to the genetic factors influencing the intercept (.14, the two components summing to .73). The model also included ACE decomposition of the time-specific residuals (i.e. percentages of variance at each age not explained by the growth factors). Supplementary Table 4 shows that e^2^ (which includes non-shared environment and measurement error) explained between 34% and 64% of the residuals. The heritability was between 36% and 58% whereas shared environment only contributed significantly at age 7 and 12 years.

### Complementary analyses

To verify whether the intercept and the slope of mother-rated conduct problems captured meaningful variance, we assessed their predictive contribution to an adolescent self-report measure of delinquency at age 16 years. In the subsample with available data (N = 1,099 pairs), both the intercept and the slope made a highly significant contribution, explaining 17% of the variance in delinquency (details in Supplementary Information).

Conduct problems include an aggressive and a non-aggressive dimension. These dimensions differ at the phenotypic level, with a regular decline in physical aggression after early childhood^16^,^17^,^18^ not necessarily paralleled by other conduct problems (e.g. stealing)^23^ Furthermore, their environmental and genetic architecture might differ. In particular, there is some evidence that heritability is stable from childhood to adolescence for aggressive antisocial behaviour whereas it increases for non-aggressive antisocial behaviour.^24^ To test whether the results were sensitive to the inclusion of both aggressive and non-aggressive problems in our conduct problems measure, we removed the aggression item from the scale and repeated the analyses. Correlations between the scale with and without the aggression item were very high, ranging from 0.95 at age 4 years to 0.98 at age 16 years. When the aggression item was removed, the linear decline in conduct problems was somewhat less steep, which is consistent with the aforementioned regular decline in physical aggression after early childhood (i.e. part of the decline was driven by the aggressive item). Genetic and environmental influences on conduct problems without the aggression item barely changed, whether in the Cholesky or in the Latent Growth models. The most important change was that the shared environmental influence on the intercept was reduced by half (from 10 to 5%) and became non-significant. Overall, our results did not appear highly sensitive to the presence or absence of aggression in the conduct problems measure (detailed results are presented Supplementary Tables 5 and 6 and Supplementary Fig. 1).

## Discussion

The aim of the study was to clarify the genetic and environmental aetiology of the developmental course of conduct problems between the ages of 4 and 16 years. Both the individual differences in the baseline level and the developmental course of conduct problems (i.e. systematic linear change) were largely explained by genetic factors. Furthermore, the genetic factors underlying the developmental course of conduct problems were largely independent from the ones underlying the baseline level. Small contributions of shared and non-shared environment were detected for the baseline level but only non-shared environment contributed to the developmental course of conduct problems.

Competing theories exist regarding the respective role of genes and the environment in behavioural development. Several, like the genetic-set point hypothesis^25^, conceptualize genes as factors of stability and, therefore, do not address directly the core finding of this research. More relevant to our results, the genetic maturation hypothesis, proposed by Lacourse *et al*.^22^, posits that systematic behavioural change in physical aggression is genetically driven by specific genetic factors that are unrelated to baseline levels of aggression. In the present study, we also found strong genetic influences on the developmental course of conduct problems, largely independent from the genetic influences affecting the baseline level of conduct problems. These findings demonstrate that, rather than only being conceptualized as factors of stability, genes also play a dynamic role in explaining systematic change in conduct problems. In other words, genetic differences to a large degree explain why some children will increase or maintain their conduct problems over time, whereas other will desist. In addition, new insights into the aetiology of change in conduct problems might be gained by integrating developmental models in molecular genetics^20^ as different sets of genes may influence the developmental course versus the baseline level of conduct problems. Indeed, a failure to integrate developmental models can leave specific genetic variants undetected, as, for example, for obesity^26^.

Insights regarding the respective nature of genetic factors influencing the baseline level and those specifically influencing the developmental course of conduct problems can be derived from Ferguson^5^. He proposed that both aggression and the control of aggression are adaptive and normative and that separate brain regions have evolved to manage aggressiveness and the control of aggression. In line with this, we speculate that a first set of genetic factors influencing the baseline level of conduct problems may be related to the temperamental make-up of the child. Negative emotionality, including anger, hostility and irritability, has been hypothesized as a key temperamental dimension underlying conduct problems^27^; other candidates are dimensions like aggressiveness, impulsivity and fearlessness^27^,^28^,^29^. A second set of genetic factors influencing the developmental course of conduct problems may relate more specifically to traits and capacities that mature in childhood and adolescence and are likely to impact upon conduct problems. As an example, sensation seeking increases during adolescence and mean-level changes in this trait are thought to be due to changes in the adolescent brain^30^. Particularly relevant is a sibling study showing that a large part of the genetic influences on the development of antisocial behaviour in adolescence were shared with genetic influences on the development of sensation seeking^31^. In addition, capacities that mature through childhood and adolescence and are relevant to conduct problems include different components of effortful control^27^ such as top-down attentional control of affect, control of impulses, and other capacities like planning, error monitoring, and decision making^32^,^33^,^34^. Such processes are important for emotion regulation, weighing the consequences of conduct problems for oneself and others, and developing strategies for engaging in alternative behaviours and thus influence the child’s propensity to persist with or desist from problem behaviour.

Contrary to Lacourse et al.^22^, in addition to a large genetic effect, we also detected a significant non-shared environmental effect on the developmental course (most probably because of a larger sample size). As such, although there is evidence of genetic maturation largely driving systematic change in conduct problems, it is not the sole explanation. The genetic maturation hypothesis^22^ can be seen as a special case of developmental effects where heritability is close to 100% and the two other dimensions are close to 0 (these two dimensions being: shared environment influences on systematic change with age or “shared change”; and non-shared environment influences that contribute to differences in systematic change with age between twins or “environmental differentiation”). Supplementary Figure 2 represents all the possible combinations between these three sources of influences on the developmental course of conduct problems.

Of interest is the absence of “shared change” in the present study. Shared environment has been shown to make small but significant and stable contributions to a large spectrum of child and adolescent psychopathological symptoms, including conduct problems^12^. Partly consistent with this finding, we detected a small and stable contribution of the shared environment from 4 years to 12 years (i.e. in the Cholesky decomposition, shared environmental factors contributing to conduct problems at 4 years were common to those accounting for variance at 7 and 12 years, whereas no shared environmental influence was present at 16 years). In addition, there was no evidence of innovation for shared environment in the Cholesky decomposition and, in the latent growth model, c^2^ only contributed significantly to the baseline level and to two time-specific residuals but not to the developmental course of conduct problems. Taken together, these findings suggest that c^2^ makes a small contribution to the stability of conduct problems in childhood but does not seem to contribute to the long-term developmental course of conduct problems. This is perhaps not surprising as twins are likely to lead increasingly independent lives as they grow older.

The present study may inform research examining environmental influences on the developmental course of conduct problems, as well as interventions designed to target those environmental influences. Research in social and behavioural sciences often does not consider genetic influences, but these findings alert us to the importance of accounting for such influences when examining the development of conduct problems. Gene-environment correlations, in particular, should be considered. For instance, bullying-victimization is associated with conduct problems and it may be tempting to conclude that being victimized is an environmental risk factor contributing to the development of later conduct problems. However, victimization is influenced in part by genetic factors^35^. This may be partly explained by evocative gene-environment correlations, whereby genetically influenced child characteristics (e.g. risk taking, aggression or conduct problems) evoke bullying responses from peers. Therefore, the correlation between victimization and conduct problems may partly reflect genetic propensities rather than an environmental main effect of victimization on conduct problems. Genetically informative designs have been used to address this issue and distinguish between: 1) so called ‘environmental influences’ that actually reflect genetic propensities and do not represent an environmental causal influence on conduct problems (e.g. maternal smoking) and 2) environmental influences that have a causal effect (e.g. maltreatment, see Jaffee et al.^36^ for a review of these designs and the predictors of antisocial behaviour). Such research is particularly informative in identifying relevant targets for intervention as it distinguishes between spurious versus likely causal environmental predictors. Furthermore, the present findings suggest that environmental influences, at least shared environmental influences, may not contribute to the long-term developmental course of conduct problems. As such, even causal environmental predictors identified in genetically informative designs may not invariably have long-term effects. Consequently, as a next step to identify relevant intervention targets, longitudinal genetically informative designs could be used to test not only if environmental factors are likely causal but, also, if their influence maintains over time. The necessity to demonstrate long-term effects holds true also for interventions targeting environmental factors to prevent the development or the maintenance of conduct problems. Longitudinal models (e.g. growth curves) can be used to test how much of the positive effect on the post-intervention status diminishes over time (i.e. positive effect on the intercept and negative effect on the slope).

The absence of shared-environmental influences on the developmental course of conduct problems in the present study may appear as a grim perspective for intervention studies targeting environmental factors. This is all the more the case as identifying risk-factors responsible for non-shared environmental effects has proven difficult^12^,^15^. These results are consistent with the challenges encountered by interventions targeting early conduct problems, as making a lasting difference has proven arduous^2^,^4^. However, heritability does not equal immutability and even highly heritable phenotypes are still amenable to intervention. Twin studies examine behaviour in a naturally occurring range of environments, and it is good to keep in mind that the estimates from these studies may not be valid under environmental constraints imposed by intervention studies (i.e. twin studies measure “what is” and not “what could be”). A thorough discussion of the multiple reasons explaining why highly heritable phenotypes can be changed is beyond the scope of this study and can be found elsewhere^15^,^22^,^37^. We want to emphasise that our results do not mean that interventions on conduct problems are bound to have short-term effects. However, they suggest that a one-time early intervention is unlikely to be a ‘magic bullet’^38^: repeated efficient interventions at different developmental stages (such as the 10-year fast tract intervention on conduct problems^39^) or enduring environmental constraints might be needed to counteract genetic propensity to conduct problems and to achieve long-term effects.

### Limitations

The measure of conduct problems, – although based on the well validated Strength and Difficulties Questionnaire widely used in clinical practice and epidemiology^8^,^40^,^41^ –yielded low internal consistency scores. Low internal consistency for this measure is not specific to this study and has been reported elsewhere^42^. Low internal consistency may inflate e^2^, which includes non-shared environment influences and measurement error. The model used partly accounted for this by having an environmental variance component for latent (relatively measurement free) as well as specific (more error capturing) factors. In the present study, e^2^ estimates for latent factors (i.e. intercept and slope) were lower than estimates reported in research using observed measures of antisocial behaviour and conduct problems^7^,^8^. The low internal consistency partly stems from the broad scope of the measure, with items covering different types of conduct problems from lying to aggression. On the one hand, this enables the measure to tap into relevant variance, which was shown by the fact that the latent factors – intercept and slope – of conduct problems were highly predictive of self-report of delinquency at age 16 years. On the other hand, this heterogeneity within the measure might have been an issue as different dimensions of conduct problems – in particular aggressive versus non-aggressive - may differ in their developmental course and their genetic/environmental architecture. However, a sensitivity analysis removing the aggression item from the conduct problems measures yielded very similar results. Finally, we relied on other reports rather than self-reports, which does influence genetic and environmental estimates, although the direction remains unclear^7^. The use of mother reports allowed us to model conduct problems starting in early childhood, when self-reports are not reliable. However, multi-informant assessment would be useful^14^.

## Conclusion

In a large sample of twins with repeated measures of conduct problems from the age of 4 to 16 years, we showed that individual differences in the developmental course of conduct problems were under strong genetic influences, different from those affecting the baseline level. In molecular studies, different sets of genes should thus be expected to be associated with the developmental course versus the baseline level of conduct problems. Furthermore, a stringent control for age in genome-wide and candidate-gene studies seems warranted as heterogeneity in age within or between samples may undermine the detection of associations and replication attempts. In addition, the structure of environmental influences on the developmental course of conduct problems indicate that repeated preventive interventions at several developmental stages might be necessary to achieve a long-term impact on preventing conduct problems. Clinicians should be aware that the maintenance of conduct problems (a decline being normative in the population) is a sign of vulnerability, independent of the baseline level. It may reflect genetic liability and warrant a closer follow-up.

## Methods

### Participants

Participants were drawn from the Twins Early Development Study (TEDS), a large longitudinal study of twin pairs recruited from population records of twin births in England and Wales between 1994 and 1996. The present study sample included a total of 10,038 twin pairs who had one or more complete (i.e. both twins) assessment(s) of conduct problems between the ages of 4 and 16 years. The comparison of the study sample with the initial contact sample and data from the United Kingdom census shows that the study sample is fairly representative of the UK population (see Supplementary Table 1 as well as additional details and references provided in the Supplementary Information). This study was approved by the Institute of Psychiatry ethics committee (consecutively 183/94; 05/Q0706/228; PNM/09/10-104) and was conducted in accordance with the ethical guidelines laid down by that committee. Parents were given a letter describing the general purpose of the study and written consent was obtained from them. It was made clear that participation was voluntary and participants could withdraw from the study whenever they wished.

### Measures

#### Conduct problems

were rated by a parent at 4, 7, 12 and 16 years, using the conduct problems scale of the Strength and Difficulty Questionnaire (SDQ), which comprises the following items: “often has temper tantrums or hot tempers”; “generally obedient, usually does what adults request,” reverse scored; “often fights with other children or bullies them”; “often lies or cheats”; “steals from home, school or elsewhere”^43^. The SDQ is widely used instrument and has been used successfully in the United Kingdom on participants aged 5 to 17 years^40^,^41^, including in a twin study of conduct problems^8^. Standardized Cronbach alphas at 4, 7, 12, 16 years were .54, .60, .62, and .65 respectively.

#### Data Analysis

All scores were regressed on sex and age prior to analyses.

#### Latent growth curve model

A latent growth curve (LGC) model was fitted to examine the developmental course of conduct problems. First, a phenotypic LGC (i.e. without genetic decomposition) was fitted to determine the baseline level (i.e. intercept) and to test whether a linear slope was sufficient to account for the observed systematic change in conduct problem or if additional growth parameters (e.g. quadratic) were necessary (detailed LGC specifications adapted for twins can be found in Olsen and Kenny^44^, Fig. 3, p.131). Second, the resulting model was modified to estimate the additive genetic (A), common or shared environment (C), and nonshared environment (E) influences on the growth factors. This ACE-LGC also enabled the estimation of how much of the genetic and environmental influences on the developmental course (i.e. slope) were shared with the baseline level (i.e. intercept). The residuals were also decomposed into ACE factors^21^ (the residual variance at each time point being the variance that is not explained by the latent factors – intercept and slope). Full Information Maximum Likelihood was used to deal with missing data and 95% confidence intervals were obtained by bootstrapping (5000 repetitions), using the bias-corrected adjusted method. For each model, we report the chi-square, the Akaike-Information Criterion and three approximate fit indexes. Details on fit indices and cut-off values used to assess model fit are provided in the Supplementary Information.

#### Software

The structural equation modelling package *lavaan 0.5-16* was used for phenotypic and biometric models^45^. All models were verified using the matrix specification package *OpenMx 1.3.2.*^46^ Packages were implemented within R software^47^ version 3.02.

## Author Contributions

Study concept and design: JBP, EV, and YZ. Statistical analyses: JBP and FR. Drafting of the manuscript: JBP, EV, and FR. Critical revision of the manuscript and approval of final version: all authors. Study supervision: FR, EV, RP. Data acquisition: EV, and RP. Obtained funding: JBP, EV, RP.

## Additional Information

**How to cite this article**: Pingault, J.-B. *et al*. Developmentally dynamic genome: Evidence of genetic influences on increases and decreases in conduct problems from early childhood to adolescence. *Sci. Rep.* **5**, 10053; doi: 10.1038/srep10053 (2015).

## Supplementary Material

Supplementary Information

## Figures and Tables

**Table 1:** MZ and DZ correlations at each age.

**Age**	**4 years**	**7 years**	**12 years**	**16 years**
MZ				
N complete pairs	2635	2715	2096	1806
Twin correlation	63 (.60−66)	75 (.72−77)	77 (.74−79)	71 (.67−74)
				
DZ				
N complete pairs	5183	4987	3723	3263
Twin correlation	33 (.30−36)	44 (.41−47)	48 (.45−52)	38 (.33−42)
N total pairs	7818	7702	5819	5069

Note. The total study sample N is superior to time specific Ns as all twin pairs having with one complete pair of data or more at one time point were included in the latent growth model (e.g. a pair of twin with missing value(s) at 4 years but available scores at 7 years was included).

**Table 2:** Cholesky decomposition of heritability, shared environment, and non-shared environment for conduct problems (CP), from age 4 years to age 16.

	**A1**	**A2**	**A3**	**A4**	**Total a**^**2**^
CP 4y	**.60(.55–.64)**				**.60(.55–.64)**
CP 7y	**.24(.19–.29)**	**.40(.34–.46)**			**.64(.57–.72)**
CP 12y	**.16(.12–.21)**	**.12(.08–.18)**	**.34(.27–.41)**		**.62(.53–.70)**
CP 16y	**.14(.10–.18)**	**.06(.04–.11)**	**.10(.05–.16)**	**.43(.37–.48)**	**.73(.69–.78)**
					
	C1	C2	C3	C4	Total c^2^
CP 4y	**.04(.01–.08)**				**.04(.01–.08)**
CP 7y	**.10(.04–.17)**	.02(.00–.08)			**.12(.06–.18)**
CP 12y	**.12(.04–.20)**	.05(.00–.13)	.00(.00–.06)		**.17(.10–.24)**
CP 16y	.01(.00–.05)	.00(–.03–.01)	.00(–.08–.00)	.00(–.03–.00)	.01(.00–.04)
					
	E1	E2	E3	E4	Total e^2^
CP 4y	**.36(.33–.38)**				**.36(.33–.38)**
CP 7y	.01(.00–.01)	**.23(.21–.25)**			**.24(.22–.26)**
CP 12y	.00(.00–.01)	.01(.00–.01)	**.20(.18–.23)**		**.21(.19–.24)**
CP 16y	.00(.00–.00)	.00(.00–.01)	.01(.01–.02)	**.24(.21–.27)**	**.26(.22–.29)**

Note. The values presented in the table are standardized components of variance. For instance, 12% of the total variance at 12 years comes from the genetic factor A2, which corresponds to age 7 years. The total c^2^ at 12 years (17%) corresponds to sum of shared environment components coming from 4, 7, 12, and 16 years. Finally, a^2^ + c^2^ + e^2^ = 1 at each age (last column, e.g. at 4 years .60 + .04 + .36). Significant estimates are in bold.
