# Supplementary material for: Developmentally dynamic genome: Evidence of genetic influences on increases and decreases in conduct problems from early childhood to adolescence
Source: Sci Rep. 2015 May 6;5:10053. doi: 10.1038/srep10053 (PMC4421862; doi:10.1038/srep10053)
Supplement: Supplementary Information [file srep10053-s1.pdf]

Supplementary Information:

Developmentally dynamic genome: Evidence of genetic influences on increases and decreases in conduct problems from early childhood to adolescence

Jean-Baptiste Pingault PhD, Frühling Rijsdijk PhD, Yao Zheng PhD, Robert Plomin PhD, Essi Viding PhD

Twins Early Development Study (TEDS): sample recruitment and description ___________ 2

Supplementary Table 1: Sample characteristics ______________________________________ 2

Supplementary Table 2: Correlation table____ 3

Supplementary Table 3: Fit indexes ____ 4

Supplementary Table 4: Decomposition of the residuals from the Latent Growth Model.___ 5

SDQ conduct problems and self-reported delinquency at age 16 years__________________ 6

Sensitivity analyses without the aggression item ____________________________________ 7

Supplementary Table 5: Conduct problems without aggression: MZ and DZ correlations at each age ____ 7

Supplementary Table 6: Conduct problems without aggression: Cholesky decomposition __ 7

Supplementary Figure 1: Genetic and environmental influences on the intercept and slope of conduct problems without aggression____ 8

Supplementary Figure 2: Three-dimensional representation of the genetic and environmental influences on behavioural development ____ 10

References Supplementary Material____ 11

Twins Early Development Study (TEDS): sample recruitment and description

The UK Office for National Statistics identified twin births between January 1994 and December 1996 through birth records, and contacted the families after screening for infant mortality. A total of 16,810 families responded to acknowledge their interest in taking part in the study. The TEDS team made their first contact with these families when the twins were about 18 month. A total of 13,722 families returned data for this first contact. eTable 1 below shows the characteristics of these respondents. Of note is that these characteristics mirror closely data from the U.K. census data, so that TEDS families appear reasonably representative of the UK population. The characteristics of the 10,038 families included in the current study sample are presented in eTable1 and match closely with first contact characteristics. Further details on the general aims of the study, the sample, data collection, and measures can be found in earlier publications.[1–4]

Supplementary Table 1: Sample characteristics

	Returned data (N families)	% White	% Mothers with A-levels or higher	% Mother employed	% Father employed	% Female	% MZ
UK census[1]	-	*93%*	*32%*	*49%*	*89%*	-	-
TEDS first contact	13,722	91.7%	35.5%	43.1%	91.6%	50.1%	33.2%
TEDS study sample	10,038	92.8%	38.9%	44.9%	92.7%	51.0%	33.8%

Note. Note. [1]UK data from the 2000 General Household Survey[5] are used rather than more recent data because they provide more appropriate comparisons for TEDS twins who were born 1994-96. The % MZ data are from Imaizumi[6] because they are not available from UK census data. A-levels are the national educational exam taken at 18 years of age in the UK, and refer to parental educational qualifications. MZ=monozygotic twins.

Supplementary Table 2: Correlation table

MZ \ DZ		TWIN 1				TWIN 2					
		4 years	7 years	12 years	16 years	4 years	7 years	12 years	16 years	MEAN (DZ)	SD (DZ)
TWIN 1	4 years	-	0.471	0.385	0.274	0.332	0.235	0.226	0.159	2.085	1.566
	7 years	0.467	-	0.500	0.388	0.244	0.442	0.276	0.172	1.715	1.648
	12 years	0.374	0.504	-	0.500	0.224	0.268	0.483	0.218	1.351	1.490
	16 years	0.280	0.333	0.455	-	0.150	0.212	0.245	0.375	1.279	1.489
TWIN 2	4 years	0.630	0.418	0.325	0.253	-	0.491	0.408	0.315	2.081	1.571
	7 years	0.410	0.749	0.460	0.303	0.467	-	0.539	0.369	1.726	1.674
	12 years	0.353	0.446	0.765	0.399	0.371	0.504	-	0.475	1.338	1.509
	16 years	0.241	0.283	0.380	0.706	0.258	0.316	0.450	-	1.260	1.401
	MEAN (MZ)	2.095	1.723	1.322	1.185	2.090	1.736	1.296	1.155		
	SD (MZ)	1.501	1.608	1.431	1.341	1.521	1.609	1.387	1.296		

Note. The table shows the observed Pearson pairwise correlations within and across time, within and between twins for MZ (lower part of the table) and DZ (upper part), as well as the observed means and standard deviations (SD). Values in grey are twin correlations at each time point: heritability and environmental estimates at each time point are based on the comparison of each pair of MZ and DZ correlations. Values in yellow represent the across time correlations for each twin, showing the phenotypic continuity in conduct problems. Values in blue represent cross-twin cross-time correlations: the respective role of genes and the environment in explaining the conduct problems is estimated based on the comparison of these correlations between MZ and DZ.

Supplementary Table 3: Fit indexes

As mentioned in the manuscript, we reported chi-square, the Akaike-Information Criterion and three approximate fit indexes for each model: CFI (Comparative Fit Index) for which values close to 1 indicate better fit; RMSEA (Root Mean Square Error of Approximation, and 90% Confidence Interval) and SRMR (Standardized Root Mean Square Residual); for the two latter, values close to 0 indicate better fit. No consensus exists on cut-off values for these indexes, but values close to .95 for CFI, 0.06 for RMSEA and 0.08 for SRMR have been suggested to conclude that there is a relatively good fit between the model and the data [7].

		AIC	Chi Square	DF	pvalue	CFI	SRMR	RMSEA	RMSEA lower	RMSEA upper
Conduct problems (CP)	Cholesky	177262	115	54	<.001	.997	.040	.015	.011	.019
	LGC phenotypic	177714	588	65	<.001	.970	.063	.040	.037	.043
	LGC ACE	177723	597	65	<.001	.970	.063	.040	.037	.043
CP without aggression item	Cholesky	164775	116	54	<.001	.996	.037	.015	.011	.019.
	LGC phenotypic	165123	486	65	<.001	.975	.060	.036	.033	.039
	LGC ACE	165138	501	65	<.001	.974	.061	.037	.034	.040

Supplementary Table 4: Decomposition of the residuals from the Latent Growth Model.

The residuals of the Latent Growth Model represent the percentages of variance at each time point that are not explained by the growth factors (intercept and slope). At each time point, the residual variance was decomposed into A (additive genetics), C (shared environment) and E (Error and non-shared environment) components. For instance, at age 4 years, the total residual variance represented 44% of the observed variance at this age (the rest being explained by the growth factors) and was mainly explained by non-shared environment and error (64% of the residual variance), the rest being explain by additive genetic effects (44%).

	Age	4 years	7 years	12 years	16 years
	Total	0.44	0.54	0.49	0.41
RESIDUALS	A	**.36 (.29-.42)**	**.51 (.38-.63)**	**.43 (.28-.56)**	**.58 (.44-66)**
	C	.00 (.00-.07)	**.12 (.02-.21)**	**.23 (.12-.34)**	.00 (.00-24)
	E	**.64 (.57-.71)**	**.38 (.33-.42)**	**.34 (.29-.39)**	**.42 (.33-50)**

SDQ conduct problems and self-reported delinquency at age 16 years

A subsample of 1,099 pairs of twins had a measure of delinquency available at age 16 years. The characteristics of this subsample were: 93.6% white, 41.2% mothers with A-levels or higher, 45.9% mothers employed, 93.4% fathers employed, 60.4% Female, 37.7% MZ. Delinquency was assessed by a self-reported questionnaire comprising 8 Yes/No items related to violent and non-violent delinquency.[8]

1. Have you ever stolen (or tried to steal) something?
2. Have you ever run away from home?
3. Have you ever attacked someone with the idea of seriously hurting or killing him or her?
4. Have you ever been involved in gang fights?
5. Have you ever hit (or threatened to hit) one of your parents?
6. Have you ever been loud, rowdy, or unruly in a public place?
7. Have you ever taken a vehicle for a drive without the owner's permission?
8. Have you ever used physical force to get money or things from a teacher or other adult at school?

These 8 items were summed to obtain the final delinquency score.

In the phenotypic latent growth curve modelling mother rated conduct problems from age 4 to age 16 years (see manuscript), the delinquency score was regressed on the intercept and the slope. Both the intercept and the slope of conduct problems significantly and independently predicted self-reported delinquency at age 16 years. The standardized estimates were 0.43 (SE: 0.04, p <.001) for the intercept and 0.42 (SE: 0.06; p <.001) for the slope. Together, the intercept and the slope predicted 17 % of the variance in the delinquency score.

Sensitivity analyses without the aggression item

Supplementary Table 5: Conduct problems without aggression: MZ and DZ correlations at each age

Age	4 years	7 years	12 years	16 years
MZ Twin correlation	.61 (.57-64)	.75 (.72-.77)	.77 (.74-.79)	.71 (.67-.74)
DZ Twin correlation	.30 (.27-.33)	.45 (.42-.47)	.48 (.45-.51)	.38 (.34-.41)

Supplementary Table 6: Conduct problems without aggression: Cholesky decomposition

	A1	A2	A3	A4	Total a^2
CP 4y	**.59 (.55-.63)**				**.59 (.55-.63)**
CP 7y	**.23 (.18-.28)**	**.39 (.33-.45)**			**.62 (.55-.69)**
CP 12y	**.16 (.12-.21)**	**.11 (.07-.17)**	**.35 (.28-.42)**		**.63 (.55-.71)**
CP 16y	**.13 (.11-.17)**	**.07 (.03-.11)**	**.10 (.05-.15)**	**.44 (.38-.49)**	**.74 (.70-.77)**
	**C1**	**C2**	**C3**	**C4**	**Total c^2**
CP 4y	**.02 (.01-.05)**				**.02 (.01-.05)**
CP 7y	**.10 (.03-.17)**	.03 (.00-.10)			**.14 (.08-.20)**
CP 12y	**.12 (.02-.20)**	.05 (.00-.13)	.00 (-.07-.00)		**.16 (.09-.23)**
CP 16y	.01 (.00-.04)	.00 (-.02-.01)	.00 (-.07-.00)	.00 (.00-.07)	.01 (.00-.02)
	**E1**	**E2**	**E3**	**E4**	**Total e^2**
CP 4y	**.39 (.36-.41)**				**.39 (.36-.41)**
CP 7y	.01 (.00-.01)	**.24 (.22-.26)**			**.24 (.22-.27)**
CP 12y	.00 (.00-.00)	.01 (.00-.01)	**.20 (.18-.23)**		**.21 (.19-.24)**
CP 16y	.00 (.00-.00)	.00 (.00-.01)	.01 (.01-.02)	**.24 (.21-.27)**	**.26 (.23-.29)**

Note. The values presented in the table are standardized components of variance. For instance, 11% of the total variance at 12 years comes from the genetic factor A2, which corresponds to age 7 years. The total c^2 at 12 years (16%) corresponds to sum of shared environment components coming from 4, 7, 12, and 16 years. Finally, $a^2 + c^2 + e^2 = 1$ at each age (last column, e.g. at 4 years .59 + .02 + .39). Significant estimates are in bold.

Supplementary Figure 1: Genetic and environmental influences on the intercept and slope of conduct problems without aggression

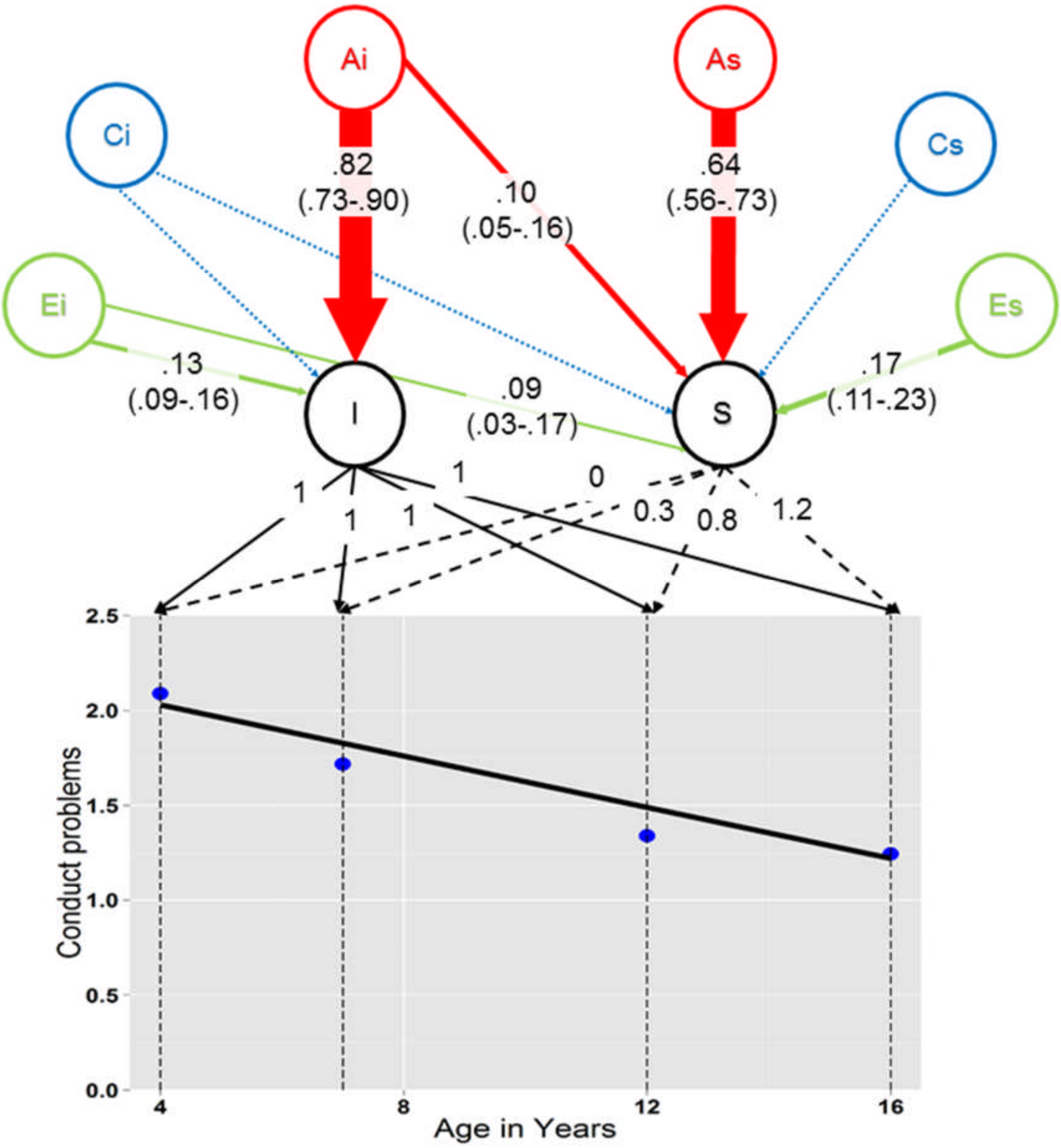

Legend e Figure1: Observed mean values of conduct problems (blue dots) and model fitted linear decrease (black line) are represented. The intercept (I) and the Slope (S) and their loadings are indicated (slope loadings equal distance in years from first measure, divided by 10 to facilitate computations). A (heritability), C (shared environment), E (non-shared environment) standardized components of variance and 95% bootstrapped confidence estimates are provided for I and S (except for the non-significant dotted lines). The width of the arrows is proportional to the effect. Dotted arrows represent non-significant effects.

Comment Supplementary Figure 1: Results for the conduct problems without the aggression item were very similar to results for the original score. The main difference is that the shared environment influence on the intercept was reduced by half and became non-significant (.05; 95% CI: .00-.13). The total genetic effect for the slope was almost identical (.74; 95% CI: .63-.85) with a total non-shared environment effects also similar (.26; 95% CI: .16-.37) and no effect of the shared environment. The figure shows that the total genetic effect on the slope was mainly due to specific effects on the slope (arrow from As to S) rather than effects shared with the intercept (arrow from Ai to S).

Supplementary Figure 2: Three-dimensional representation of the genetic and environmental influences on behavioural development

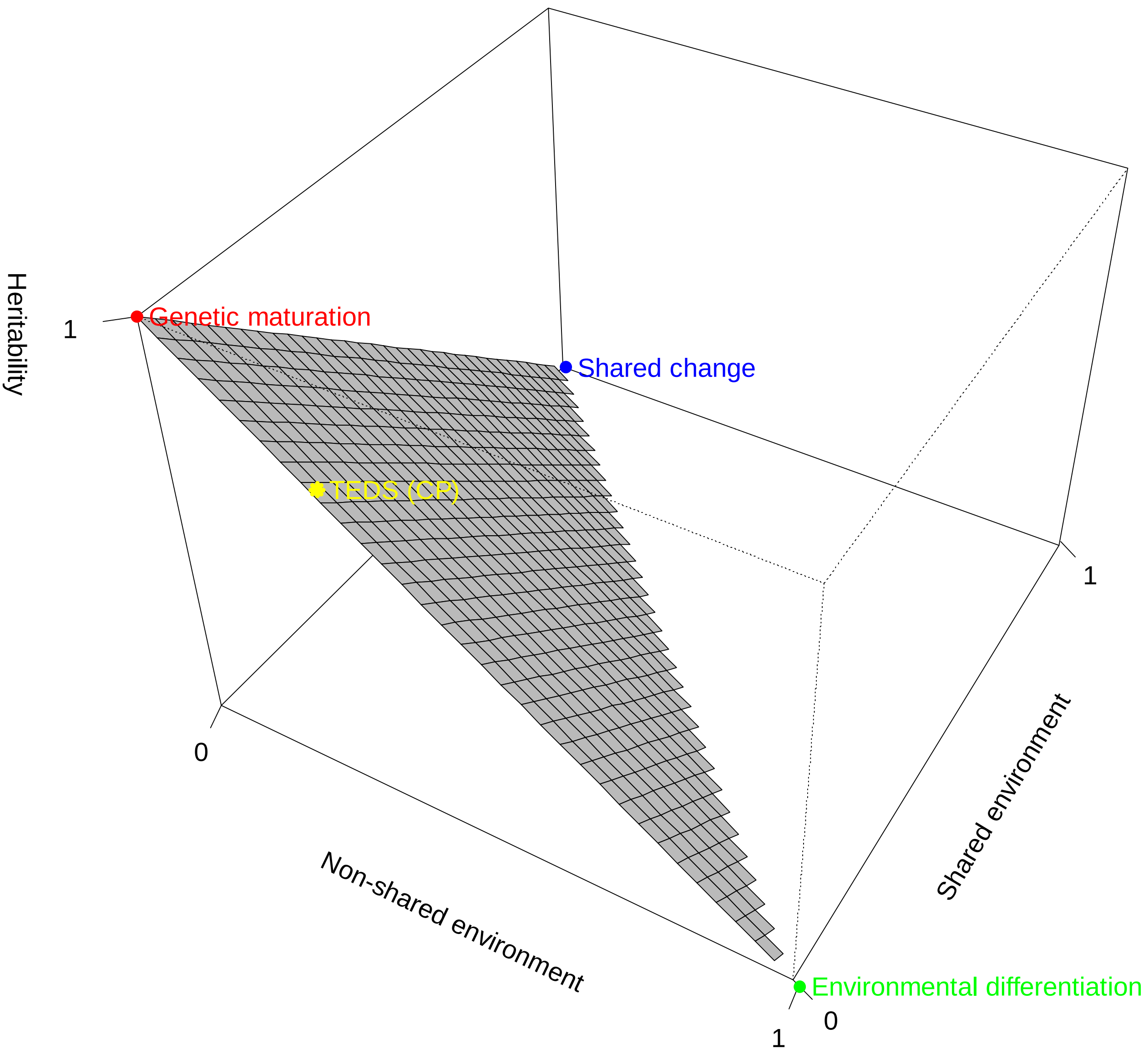

Legend: The grey plane draws $a^2 + c^2 + e^2 = 1$, thus representing all the possible combinations of genetic (a^2), shared (c^2) and non-shared environmental (e^2) influences for a given phenotype, applied here to the developmental course (e.g. linear slope in the present study). "TEDS (CP)", in yellow, represents the significant influences on the linear slope of conduct problems in the present study (heritability = 0.73 & non-shared environment = 0.25). The "genetic maturation" hypothesis is a special case of developmental effect (heritability = 1, the rest = 0). "Shared change" and "Environmental differentiation" represent the other extreme cases of developmental effects.

References Supplementary Material

1 Kovas Y, Haworth CMA, Dale PS, Plomin R. The genetic and environmental origins of learning abilities and disabilities in the early school years. *Monogr Soc Res Child Dev* 2007; **72**: vii, 1–144.

2 Oliver BR, Plomin R. Twins' Early Development Study (TEDS): a multivariate, longitudinal genetic investigation of language, cognition and behavior problems from childhood through adolescence. *Twin Res Hum Genet* 2007; **10**: 96–105.

3 Trouton A, Spinath FM, Plomin R. Twins early development study (TEDS): a multivariate, longitudinal genetic investigation of language, cognition and behavior problems in childhood. *Twin Res Off J Int Soc Twin Stud* 2002; **5**: 444–448.

4 Haworth CMA, Davis OSP, Plomin R. Twins Early Development Study (TEDS): a genetically sensitive investigation of cognitive and behavioral development from childhood to young adulthood. *Twin Res Hum Genet Off J Int Soc Twin Stud* 2013; **16**: 117–125.

5 ONS. *Living in Britain: Results from the 2000 General Household Survey*. Stationery Office: London, 2001.

6 Imaizumi Y. A comparative study of zygotic twinning and triplet rates in eight countries, 1972-1999. *J Biosoc Sci* 2003; **35**: 287–302.

7 Hu L, Bentler PM. Cutoff criteria for fit indexes in covariance structure analysis: Conventional criteria versus new alternatives. *Struct Equ Model Multidiscip J* 1999; **6**: 1–55.

8 Elliott D, Huizinga D, Ageton S. Explaining delinquency and drug use. Sage Publications: Beverly Hills, California, 1985.
